# Role of the Blood-Brain Barrier in Central Nervous System Insulin Resistance

**DOI:** 10.3389/fnins.2019.00521

**Published:** 2019-06-04

**Authors:** Elizabeth M. Rhea, William A. Banks

**Affiliations:** ^1^Geriatric Research Education and Clinical Center, Veterans Affairs Puget Sound Health Care System, Seattle, WA, United States; ^2^Division of Gerontology and Geriatric Medicine, Department of Medicine, University of Washington, Seattle, WA, United States

**Keywords:** insulin, transport, blood-brain barrier, neurovascular unit, insulin resistance

## Abstract

The blood-brain barrier (BBB) mediates the communication between the periphery and the central nervous system (CNS). Recently, CNS insulin resistance has been elucidated to play a role in neurodegenerative disease. This has stimulated a wealth of information on the molecular impact of insulin in the brain, particularly in the improvement of cognition. Since the BBB regulates the transport of insulin into the brain and thus, helps to regulate CNS levels, alterations in the BBB response to insulin could impact CNS insulin resistance. In this review, we summarize the effect of insulin on some of the cell types that make up the BBB, including endothelial cells, neurons, astrocytes, and pericytes. We broadly discuss how these changes in specific cell types could ultimately impact the BBB. We also summarize how insulin can regulate levels of the pathological hallmarks of Alzheimer’s disease, including amyloid beta (Aβ) and tau within each cell type. Finally, we suggest interventional approaches to overcome detrimental effects on the BBB in regards to changes in insulin transport.

## Introduction to the Blood-Brain Barrier

The blood-brain barrier (BBB) is a component of the neurovascular unit (NVU) and acts as the blood-brain interface, mediating communication between the central nervous system (CNS) and the periphery. The BBB separates the circulation from the brain, allowing for protection from and transport regulation of serum factors and neurotoxins. The BBB is not just a physical barrier (due to the presence of specialized tight junctions and other changes that prevent unregulated leakage) but also acts more selectively as a transport interface (with specific transporters present on luminal and abluminal membranes), a secretory body, and a metabolic barrier (containing and releasing certain enzymes locally) (Abbott et al., 2006).

## Cells Forming the Blood-Brain Barrier

We introduce the cells and structures that make up the BBB here so we can discuss insulin receptor signaling in each of them later. For more detailed reviews of these BBB components, we refer the reader to the following excellent reviews (Broadwell, 1989; Begley, 2004; Neuwelt et al., 2008; Banks, 2016; Sweeney et al., 2019). It is important to keep in mind that the BBB is not the same in all regions throughout the brain. Therefore, observations in one brain region or subregion might not persist in another. The same will hold true for insulin signaling and insulin transport. The diameter of blood vessels and composition of the BBB can change depending on the requirements of the region and the type of blood vessel (i.e., arterioles to capillaries to venules). We do not discuss other components of the NVU including the extracellular matrix, vascular smooth muscle cells, and other glia cells due to limited available information on insulin signaling. However, with growing interest in CNS insulin signaling and the impact both centrally and peripherally, we expect further research endeavors exploring these other components of the NVU.

### Brain Endothelial Cells

The first line of defense against circulating factors for the brain is a monolayer of brain endothelial cells which are connected to one another by tight junctions and adherens junctions (Tietz and Engelhardt, 2015). These endothelial cells are different from peripheral endothelial cells in that they express tight junction proteins, creating a stronger barrier, and have decreased pinocytosis, restricting vesicle-mediated transcellular transport and transporters (Reese and Karnovsky, 1967). They make up the largest surface area at the blood-CNS interface. With this large surface area, they can readily transport proteins and molecules into and out of the brain most efficiently. Endothelial cells are polarized, exhibiting a luminal and abluminal side, with different transporters and cellular machinery expressed at each side. While this specific cell type is most often modeled as the BBB *in vitro*, there are other cell types present that are a part of the NVU or that affect BBB functions, including neurons, astrocytes, and pericytes.

### Neurons

Neurons remain close to the capillaries and connect with astrocytic endfeet in near proximity to the BBB. Neurons are rarely more than 8–20 μm from a brain capillary (Schlageter et al., 1999). It is estimated that nearly each neuron has its own capillary (Zlokovic, 2005). The close proximity to the endothelial cells, allows neurons to respond to the ever changing local milieu, especially in regards to ion balance. Neurons play a role in regulating blood flow, microvascular permeability, interact with the extracellular matrix, and can release factors to stimulate angiogenesis (Zlokovic, 2008). Following a vascular insult, signals from neurons and astrocytes can recruit microglia which secrete proinflammatory cytokines (Man et al., 2007). Neurons help tighten brain endothelial cells in culture by aiding in tight junction protein synthesis and localization (Savettieri et al., 2000). These data support a synergistic role for the regulation of other cell types by neurons and highlight how these cells communicate with one another. Indeed, the neuronal circuitry is linked to the blood vessels by water channels present in astrocytes.

### Astrocytes

Astrocytes are the most abundant cells in the brain, providing an environment to help regulate all aspects of neuronal function (survival, development, metabolism, neurotransmission). They act as metabolic sensors in the brain responding to changes in the local environment (Garcia-Caceres et al., 2016). At the BBB, astrocytes help provide maintenance and repair support through release of several effector molecules (Wosik et al., 2007; Alvarez et al., 2011; Bell et al., 2012). The astrocytic endfeet ensheath the vascular tube and help to regulate ion and water regulation (Abbott et al., 2006). Aquaporin-4 is an astroglial water channel that regulates perivascular fluid and solute movement through the glymphatic system, a unique exchange between perivascular cerebrospinal fluid (CSF) and interstitial fluid present in the CNS (Iliff et al., 2012; Nedergaard, 2013; Mestre et al., 2018). Using this system, the brain can regulate fluid flow throughout the CNS and aid in clearance of toxins. In addition, the connection between neurons and blood vessels allows astrocytes to relay signals regarding blood flow (Hamilton and Attwell, 2010) as well as controlling brain water content (Zlokovic, 2008). Of the approximately 11 distinct phenotypes of astrocytes, 8 are involved in interactions with blood vessels (Reichenbach and Wolburg, 2005; Abbott et al., 2006). Astrocytes and endothelial cells have a symbiotic relation. Astrocytes secrete a range of chemical factors, including various growth factors that induce aspects of the BBB phenotype in endothelial cells *in vitro* and likely *in vivo* while endothelial cells aid in astrocytic differentiation (Mi et al., 2001; Lee et al., 2003; Abbott et al., 2006). Astrocytic end feet are polarized and guided to cerebral vessel walls by pericytes (Armulik et al., 2010).

### Pericytes

Pericytes sit on the abluminal surface of the endothelial cell and are embedded in the vascular basement membrane and are physically connected to brain endothelial cells by way of gap junctions and peg and socket arrangements (Miller and Sims, 1986). Pericytes help to maintain and stabilize the monolayer of brain endothelial cells by regulating angiogenesis and depositing extracellular matrix. Pericytes are essential for development of tight junctions, including in the development of barrier functions in utero (Daneman et al., 2010; Hayden et al., 2013). In addition, there is cross talk from the brain endothelial cell to the pericyte on pericyte proliferation and migration. CNS pericytes also have distinct properties from their peripheral counterparts. The endothelial:pericyte ratio is much greater in the CNS, estimated to be about 4:1 in mice (Bonkowski et al., 2011), compared to other tissues which have just one pericyte per 100 endothelial cells (Shepro and Morel, 1993). Pericytes can regulate blood flow in response to neural activity (Armulik et al., 2010; Daneman and Prat, 2015) suggesting an important role in mediating vascular tone and highlighting the neural communication necessary for this particular function.

These cell types (brain endothelial cells, neurons, astrocytes, and pericytes) communicate with one another to not only help form the BBB but also to regulate its structure and function. As touched on above, these cells can communicate with secretory factors in addition to changes in fluid movement and water channels. Interruptions in signaling within one cell type could have detrimental effects in all cell types. For example, pericyte loss has been shown to occur in some animal models of peripheral insulin resistance (Price et al., 2012; Salameh et al., 2016; Warmke et al., 2016) and are one of the first cell types of the BBB to degenerate in Alzheimer’s disease (Sengillo et al., 2013). Loss of pericytes can lead to BBB breakdown, causing a dysfunction in the transport regulation of blood-to-brain and brain-to-blood factors. Pericyte loss accelerates development of Alzheimer’s disease pathology including amyloid beta (Aβ) deposition, tau pathology, and neuronal loss (Sagare et al., 2013). In the next section, we will describe the role of the insulin receptor in each of these cell types and speculate how insulin resistance in one cell type might adversely affect some of the other BBB cell types.

## Insulin Signaling Within Cells of the Blood-Brain Barrier

**FIGURE 1:**
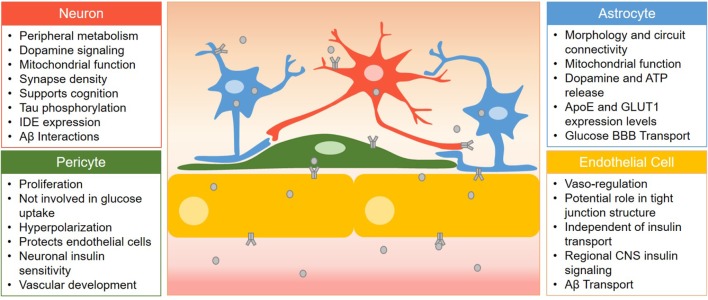
Summary of the role of insulin receptor signaling in various cells of the BBB. Cell types are listed here with the role of insulin receptor signaling listed in bullet points. Insulin (gray circles) must first cross barrier cells in order to activate insulin receptors (gray receptor) in the CNS located on neurons, astrocytes, and pericytes.

There is not a cell type in the CNS that we are aware of that does not express the insulin receptor. In mice, the expression of the insulin receptor gene is most abundant in endothelial cells, about two times greater than astrocytes, with neurons falling in close behind in terms of RNA expression levels^1^ (Zhang et al., 2014). This same expression pattern was not observed in samples from human tissue (Zhang et al., 2016). Instead, expression of the insulin receptor is more evenly distributed between the cell types. Insulin interacts with receptors on neurons and glial cells (Unger et al., 1989), endothelial cells (Konishi et al., 2017; Rhea et al., 2018), and pericytes (Sweeney et al., 2016) to elicit various physiological effects, some of which are highlighted in Figure 1. The insulin receptor exists in two isoforms, an A and B form, due to differences in splicing of the α subunit, resulting in different binding affinities to insulin and insulin-like growth factor (Belfiore et al., 2017). However, until recently, the ability to detect these two isoforms by immunological methods *in vivo* in different cell types has been a challenge. With the advances in single cell RNA sequencing (Ofengeim et al., 2017) and a novel *in situ* RT-PCR/FISH assay (Spencer et al., 2018), we expect a growth in the knowledge of expression pattern of these isoforms and alterations in human disease within specific cell types and regional variations. The insulin receptor can also form heterodimers with the IGF-1 receptor and can have varying post-translational modifications leading to further diversity of insulin action (Wozniak et al., 1993; Chiu and Cline, 2010).

Figure 1 summarizes the role of the insulin receptor on each BBB cell type discussed in this review. Various groups have utilized Cre-loxP-mediated recombination (Gu et al., 1994) to generate cell specific knock-out models of the insulin receptor to investigate the impact of disrupted insulin signaling. We have included a table listing the studies generating and utilizing some of these CNS insulin receptor knock-out models (Table 1). This section describes the importance of the insulin receptor in components of the BBB from research generated primarily in these knock-out mice with a focus not only on organization of the BBB but also on Alzheimer’s disease pathology and cognition.

**Table 1:** Summary of the generation of CNS insulin receptor knock-out models relating to the BBB.

Cell type	Model	Cre driver	References
Endothelial Cell	VENIRKO	*Tie2*	Vicent et al., 2003; Kondo et al., 2004
	EndoIRKO	*Cdh5*	Konishi et al., 2017; Rhea et al., 2018
Neuron	NIRKO	*Nestin*	Bruning et al., 2000; Schubert et al., 2004; Kleinridders et al., 2015
	nIR -/-	*Synapsin*	Freude et al., 2009; Stohr et al., 2013
Astrocyte	GIRKO	*GFAP*	Garcia-Caceres et al., 2016; Cai et al., 2018
Pericyte	PIR -/-	*PDGFRβ2*	Warmke et al., 2017


*These references highlight the critical work investigating the role of the insulin receptor on BBB function in addition to memory impairments in CNS insulin resistance.*

### Brain Endothelial Cell

Mice lacking the insulin receptor on vascular endothelial cells (VENIRKO) were first generated in Vicent et al. (2003). The original study introducing these mice investigated the role of the endothelial cell in regulating vascular tone and peripheral insulin resistance. Cerebrovascular microvessels and endothelial cells from the lungs were isolated and cultured to measure the effectiveness of the knock-out (which resulted in 95–97% decrease in mRNA levels). Abnormal architecture of capillary integrity could not be detected in brain (Vicent et al., 2003). Using a euglycemic-hyperinsulinemic clamp, the authors found endothelial cell insulin receptors do not play a role in the access of insulin to peripheral metabolically active tissues but did not investigate brain. Loss of the endothelial insulin receptor resulted in decreased levels of eNOS, an important regulator of vascular tone which could affect exposure to various circulatory factors. Indeed, the cerebrovascular response to insulin appears to be biphasic with vasoconstriction at low doses and vasodilation at higher doses (Katakam et al., 2009). Vascular integrity of the BBB was later investigated in these mice (Kondo et al., 2004). There was no disruption to Evans blue in the hemispheres or cerebellum in VENIRKO mice compared to littermate controls. Levels of ZO-1, a tight junction protein, were unchanged in addition to levels of astrocytes, as measured by GFAP staining. However, in another model of insulin receptor loss in endothelial cells (EndoIRKO), permeability of the BBB to a 3 kDa dextran was increased in the olfactory bulb and median eminence compared to controls and ZO-1 was decreased by 40% in the hypothalamus (Konishi et al., 2017). These differences between the two models could be due to regional regulation of tight junction proteins by the insulin receptor. Using the EndoIRKO model, it was demonstrated insulin receptors on brain endothelial cells control the kinetics of insulin signaling in certain regions of the brain, such as the hippocampus and hypothalamus, but not the olfactory bulb (Konishi et al., 2017). Decreased binding of insulin was observed in the frontal cortex endothelium of EndoIRKO mice. The results from these two models suggests the insulin receptor in endothelial cells has a regional effect in relaying insulin signaling to other cell types in addition to maintaining the BBB structure by regulating tight junction protein expression.

In addition to the genetically modified mouse models, some groups have utilized a selective inhibitor of the insulin receptor, S961, *in vitro* to investigate the role of the brain endothelial cell insulin receptor. This inhibitor has high affinity and selectivity, especially over the IGF-1 receptor (Schaffer et al., 2008). Insulin binding was decreased with S961 treatment (Hersom et al., 2018) and insulin-induced phosphorylation of Akt was decreased with S961 treatment (Gray et al., 2017; Hersom et al., 2018). It was also shown that while the downstream insulin receptor signaling mediator PI3K was inhibited, insulin uptake was not altered (Gray et al., 2017). On the other hand, high-fat diet decreased insulin uptake, yet insulin receptor signaling was unaltered (Gray et al., 2017). These data suggest a disconnect between insulin receptor signaling and insulin transport across the endothelial cell which we will discuss later.

Impaired brain clearance of Aβ across the BBB is thought to be part of the contribution of Alzheimer’s disease pathogenesis. Insulin can regulate Aβ trafficking at the BBB in both the luminal-to-abluminal and abluminal-to-luminal directions (Vandal et al., 2014; Swaminathan et al., 2018). Systemic insulin increased Aβ40 plasma clearance and transport from blood-to-brain but decreased Aβ42 (Swaminathan et al., 2018). On the other hand, brain Aβ40 BBB clearance is decreased while Aβ42, the more toxic and amyloidogenic form, is increased after insulin treatment. If insulin is administered intracerebroventricularly, Aβ40 clearance is inhibited (Shiiki et al., 2004). Therefore, it is important to consider the effects of insulin at the BBB when administered luminally versus abluminally. The impact of insulin on Aβ transport at the BBB has also been investigated in obese mice in the triple-transgenic model of Alzheimer’s disease (3xTg-AD) (Vandal et al., 2014). An acute intravenous injection of insulin (5 min before sacrifice) restored cortical soluble Aβ40 and Aβ42 back to the level of 3xTg-Ad mice fed a control diet. There was a concomitant increase in plasma Aβ following insulin injection. In two separate human studies, it was found plasma Aβ42 levels increased following insulin administration (Kulstad et al., 2006; Karczewska-Kupczewska et al., 2013). These studies suggest BBB clearance is likely the process by which insulin decreases brain Aβ levels. Based on the density of the cerebravasculature throughout the brain, it is possible that Aβ can be excreted rapidly out of the brain with the widespread network of capillaries, venules and veins.

### Neurons

Insulin signaling in the CNS is important for promoting neuronal survival and regulating key processes involved in learning and memory (synapse density, plasticity, and connectivity). Insulin in the brain is more closely linked to its ancestral roles by acting more as a growth factor rather than acting as a metabolite to regulate glucose uptake as occurs in the periphery (Banks et al., 2012). Peripheral injection of 1 mU insulin increased cerebral insulin signaling within 5 min, which was localized to the plasma membrane of a subset of neurons (Freude et al., 2005).

Mice lacking the insulin receptor specifically in the brain (NIRKO) were generated in Bruning et al. (2000). NIRKO mice have a 95% reduction in total insulin receptor expression in brain with no detectable change in peripheral tissues such as skeletal muscle, heart, and liver. The loss of the insulin receptor leads to impaired peripheral metabolism as the mice aged. NIRKO mice exhibit decreased dopamine signaling and impairments in mitochondrial function (Kleinridders et al., 2015). In *Xenopus* tadpoles, loss of the insulin receptor specifically in tectal neurons reduces synapse density, decreases activation, and alters morphology (Chiu et al., 2008).

Young and aged NIRKO mice do not have memory impairments compared to age-matched controls (Schubert et al., 2004). In addition, the insulin receptor was not required for neuronal survival *in vivo* (Bruning et al., 2000). While this data is not in line with other reports on the mechanism of insulin in the CNS to promote neuronal survival and play a role in memory, it is likely compensation has occurred due to complete loss of the insulin receptor throughout the brain for the entire life of the animal, as previously suggested (Grillo et al., 2015). If the insulin receptor is downregulated specifically in the hippocampus of adult rats using a lentivirus, long-term memory is impaired (Grillo et al., 2015). It is important to note that there were no metabolic or endocrine changes with specific knock-down in the hippocampus despite prior work by this group showing metabolic changes when targeted to the hypothalamus (Grillo et al., 2007). These differences when the insulin receptor is targeted in specific regions highlight the need to further investigate the role of the insulin receptor in a regional context.

Loss of the insulin receptor increases phosphorylation of tau, suggesting CNS insulin resistance could lead to an increase in this pathological mediator of Alzheimer’s disease. In addition, the insulin stimulated phosphorylation of tau was at a site demonstrated to form tangles (Kimura et al., 1996). This effect of insulin on tau phosphorylation seems to be time dependent based on studies *in vitro* (Lesort et al., 1999; Lesort and Johnson, 2000). Tau phosphorylation was completely abolished in NIRKO mice (Schubert et al., 2004). These data show that insulin receptor signaling can impact tau phosphorylation given the right time and environment.

While numerous groups have shown insulin can affect tau phosphorylation, there are fewer studies suggesting tau can regulate insulin signaling. Insulin accumulates intraneuronally together with hyperphosphorylated tau in Alzheimer’s disease (Rodriguez-Rodriguez et al., 2017). Tau pathology triggers insulin accumulation and oligomerization. Inhibition of tau phosphorylation using okadaic acid decreased insulin receptor expression levels in neurons, and this was dependent on the presence of extracellular insulin. In addition, neurons with increased tau hyperphosphorylation have enhanced insulin uptake (Rodriguez-Rodriguez et al., 2017).

Primary neuronal cultures with dysfunctional insulin receptor (transfected with kinase dead insulin receptor) have increased levels of Aβ oligomers and exacerbated aggregation (Zhao et al., 2009). It is thought that stimulation of the insulin receptor in neurons activates insulin degrading enzyme (IDE), reducing the risk of Aβ buildup. Indeed, activation of insulin receptor signaling enhances IDE expression in neurons (Zhao et al., 2004). Insulin can protect cultured rat hippocampal neurons from Aβ-induced toxicity (Takadera et al., 1993). In addition, Aβ competes with insulin in binding the insulin receptor, decreasing the phosphorylation and thus, activity of the insulin receptor (Xie et al., 2002). Because these studies were completed with purified components (i.e., insulin receptor isolated from plasma membranes, insulin, and Aβ), it was only later shown by Rensink et al. (2004) *in vitro* that insulin interacted with Aβ directly to limit membrane binding and fibril formation (see pericyte section). In addition, Aβ oligomers have been shown to lead to the removal of insulin receptors from the membranes of neuronal processes (Zhao et al., 2008; De Felice et al., 2009). Neuronal specific knockout of the insulin receptor using a *synapsin-1* Cre driver reduced Aβ (1–40 and 1–42) accumulation in the Tg2576 Alzheimer’s disease mouse model (Stohr et al., 2013). IDE preferentially degrades insulin over Aβ (Qiu et al., 1998) suggesting levels of insulin can regulate levels of Aβ. Lastly, gene expression of amyloid processing genes, such as APP and PSEN1/2, have varying degrees of correlation with expression levels of the insulin receptor and IRS1 within the brain and are considered “co-expressed” genes that share similar spatial expression patterns (Diehl et al., 2017). These results greatly support the interaction of insulin, the insulin receptor, and Aβ within neurons linking changes in CNS insulin with the development of AD pathology. However, it is still unclear whether disrupted insulin receptor signaling leads to alterations in Aβ clearance and degradation or whether Aβ accumulation leads to disruptions in insulin receptor signaling.

### Astrocytes

Primary human astrocytes express the insulin receptor and downstream signaling mediators and are responsive to insulin by altering glycogen synthesis and cell proliferation (Heni et al., 2011). Astrocytes can respond to insulin concentrations as low as 1 nM, concentrations that are commonly exceeded in the blood of healthy humans following feeding. These data suggest that even if CSF insulin levels are low, astrocytes might be exposed to comparable blood levels due to their close contact with blood vessels. Specifically in astrocytes, insulin receptor gene expression increases with age in the mouse (Clarke et al., 2018). By 9.5 months in mice, levels have reached their highest in the cortex and striatum. Interestingly, it is not until the age of 2 years that mouse hippocampal astrocytes reach their peak in insulin receptor gene expression. Astrocytes are one type of brain cell that have the ability to proliferate in adults. Astrocyte cell numbers increase after addition of insulin to the culture medium but high glucose inhibits astrocyte proliferation (Li et al., 2018). Astrocytes predominantly express the insulin receptor-B isoform (Garwood et al., 2015).

While the loss of insulin receptor signaling in neurons has been studied for decades, the loss in astrocytes has only recently begun to be investigated. Astrocyte insulin receptor knock-out mice (GIRKO) have been generated recently (Garcia-Caceres et al., 2016; Cai et al., 2018). Insulin receptor levels in astrocytes were decreased by about 50–70% (Garcia-Caceres et al., 2016; Cai et al., 2018). Postnatal loss of the insulin receptor in astrocytes affects morphology, circuit connectivity, and mitochondrial function (Garcia-Caceres et al., 2016). Insulin signaling in astrocytes also plays a role in potentiating release of dopamine and ATP (Cai et al., 2018). In an insulin-deficient mouse model of diabetes, astrocytes retract at the BBB (Salameh et al., 2016). This ultimately leads to a disruption of the BBB, both structurally and via permeability to sucrose. BBB permeability in models lacking the astrocytic insulin receptor has not been investigated. The loss of the insulin receptor reduces activation of neurons by glucose, which ultimately alters glucose transport across the BBB (Garcia-Caceres et al., 2016). GIRKO mice have decreased expression of GLUT1 and shifts fuel preference of astrocytes from glucose to lipids. GLUT1 is more abundantly expressed in astrocytes compared to brain endothelial cells (Simpson et al., 1999). Decreased GLUT1 due to loss of astrocyte insulin receptor likely decreases glucose transport across the BBB. Alterations in GLUT1 expression at the BBB is associated with Alzheimer’s disease (Winkler et al., 2015). Astrocytes are a major source of apolipoprotein E (apoE) in the brain (Kim et al., 2009). The secretion of apoE4 from astrocytes led to impaired barrier function *in vitro* (Nishitsuji et al., 2011) which has been implicated in Alzheimer’s disease. Loss of the insulin receptor increased apoE expression by approximately 40% in astrocytes (Cai et al., 2018). This data suggests insulin can regulate multiple aspects of astrocyte function, which can ultimately affect neuronal plasticity and activity in the brain.

In humans, systemically administered insulin can increase CSF Aβ42 levels (Watson and Craft, 2003). It is possible the aquaporin-4 water channels present in astrocytes can aid in the insulin-dependent BBB clearance of CNS Aβ levels either through BBB clearance or by increasing CSF turnover to shuttle Aβ out through the glymphatic system (Vandal et al., 2015). Loss of the insulin receptor present in astrocytes or impairment in insulin response could weaken this clearance.

### Pericytes

Human pericytes express the insulin receptor (James and Cotlier, 1983) yet the alpha subunit is undetectable in cultured human brain pericytes (Rensink et al., 2004). Insulin does not stimulate glucose uptake in cultured human brain pericytes (Rensink et al., 2004) or retinal capillary pericytes (Mandarino et al., 1994). It has also been shown that cell proliferation is enhanced more in pericytes due to insulin exposure compared to endothelial cells (King et al., 1983). Most studies investigating the role of the insulin receptor in pericytes have been done on cells isolated from bovine retinal capillaries (Escudero et al., 2017). Insulin can induce hyperpolarization of pericytes through calcium sensitive potassium channels (Berweck et al., 1993). Pericyte insulin signaling reduces endothelial cell death (Kobayashi and Puro, 2007). Pericyte-derived media, but not astrocyte-derived media, was able to increase the insulin stimulated phosphorylation of Akt and insulin receptor in a hypothalamic neuronal cell line, suggesting pericytes can increase insulin sensitivity in these neurons (Takahashi et al., 2015).

A mouse model with a pericyte specific knockout of the insulin receptor was used to investigate the role of insulin signaling in retinal angiogenesis (Warmke et al., 2017). Early on (postnatal day 5), retinas are hypervascularized in the knockout mice, which did not persist into adulthood. Alternatively, while pericyte coverage was similar between controls and knock-outs at this age, pericyte levels were reduced by 20% in the adult retinal vasculature. Changes in insulin signaling, pericyte function, or BBB changes were not reported in this abstract. In addition, PDGFRβ2, a marker commonly used for pericyte specificity, is expressed in other mural cells including vascular smooth muscle cells which would delete the insulin receptor in these cells as well.

Insulin can protect primary human brain pericytes from the toxic nature of Aβ (Rensink et al., 2004). Insulin inhibits Aβ fibril formation, binding of Aβ to the cell surface, and potentially interacts with Aβ itself (Rensink et al., 2004). Therefore, the protective effects of insulin in Alzheimer’s disease could be due to pericyte protection from Aβ toxicity.

Even though the loss of the insulin receptor in specific cell types of the NVU has not been exhaustively investigated within the last two decades, there is still much to learn from these various models. For example, it is largely unknown what combinatorial effects might occur due to the loss of the insulin receptor in multiple cell types. Second, it is largely unknown how the loss of the insulin receptor in one cell type impacts another cell type. The use of the novel *ex vivo* technique utilizing BBB organoids (Bergmann et al., 2018) in addition to *in vitro* co-culture experiments could help answer some of these questions about basic interactions. Third, the regional effect of the insulin receptor has hardly been studied. More studies utilizing targeted knock-down of the insulin receptor should be performed in order to better understand the specific role of the insulin receptor in regions dedicated to different processes. Another way to get at this question would be to utilize optogenetics to inhibit the insulin receptor in certain sub-populations of cell types to determine the downstream impact. Fourth, as a recent study suggests that 25% of the neurons present in the adult human frontal cortex does not express the insulin receptor (Spencer et al., 2018), it will be important to also understand why insulin receptor signaling might not be necessary in this rather large subset of cells. Lastly, something that has not really been touched on here but is important to consider is the location of the insulin receptor within the cell types. It has been shown in cultured hippocampal neurons that the insulin receptor is present primarily in the postsynaptic density (Abbott et al., 1999), suggesting a role for insulin receptor signaling in mediating communication between neurons. However, the localization of the insulin receptor in other cell types in other regions has largely been uninvestigated. The molecular impact of the insulin receptor present within these cell types of the BBB is gaining great interest, likely due to the link in CNS insulin resistance and Alzheimer’s disease.

## Insulin Transport at the BBB

Investigators have been examining the transport of insulin into the brain since 1954 when it was observed that minimal amounts of radioactively labeled insulin appeared in brain tissue following intravenous or subcutaneous injection (Haugaard et al., 1954). It was later more definitively shown that serum insulin appeared in CSF in dogs following insulin infusion (Margolis and Altszuler, 1967). Intravenously administered insulin is detected in brain within 1 min (Banks and Kastin, 1998; Banks et al., 1999). Transport of insulin across the BBB has been validated many times using various techniques including perfusions (Schwartz et al., 1991), species-specific immunoassays (Banks et al., 1997c), and state-of-the art kinetic analyses (Banks et al., 1997a). The transporter for insulin at the BBB is not static but rather a dynamic protein regulated by the current physiological state of the body. In fact, during a time in which the brain is developing the greatest, the neonatal period, insulin transport across the BBB and binding to the brain endothelial cells is increased compared to weanling and adult periods (Frank et al., 1985). CSF and brain insulin levels are also significantly greater in the neonatal period. Insulin binding to brain capillaries is highest in the newborn rabbits compared to adults suggesting the presence of higher levels of insulin binding sites (Frank et al., 1985). These discrepancies between neonates and adults is likely due to the mitogenic nature of insulin action in the CNS rather than the metabolic role. Other physiological regulators of insulin transport that relate to insulin resistance are discussed in the next section.

**FIGURE 2:**
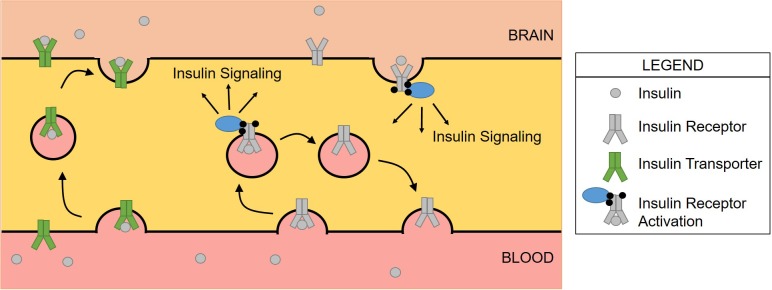
Schematic of insulin transport versus insulin receptor signaling at the BBB. Insulin binds the insulin receptor where the complex is internalized via endocytosis. Insulin activates the receptor, triggering a signaling cascade before insulin then dissociates from the receptor. The receptor is then recycled to the membrane for subsequent signaling. The insulin transporter is responsible for delivering insulin from the blood to the brain, navigating across the brain endothelial cell. This has been demonstrated in only the luminal to abluminal direction suggesting a modification of the insulin transporter that prevents binding on the abluminal surface.

We also know the rate of transport of insulin BBB transport varies between brain regions based on requirement. Insulin transport is not flow dependent like glucose. For some time now, it has been thought the insulin receptor present on the brain endothelial cell mediates this transport. This concept has some validity to it as regions in which insulin receptor expression is greatest, such as the olfactory bulb (Schulingkamp et al., 2000; Ghasemi et al., 2013), transport is greatest (Banks et al., 1999; Rhea et al., 2018). However, what is not necessarily taken into consideration is the amount of insulin receptor present on neurons, astrocytes, pericytes, and other CNS cell types in these brain regions versus the levels present on brain endothelial cells. With the increasing use of single-cell RNA sequencing, we are beginning to learn more about the expression pattern of the insulin receptor in different CNS cell types (Zhang et al., 2014) and will further be able to identify regional differences between the cell types. In addition, variable cerebral blood circulation, diverse capillary density in the brain, or other factors, such as expression levels of insulin transport protein mediators could also drive the regional transport differences. Studies using *in vitro* transport models (Gray et al., 2017), capillary binding assays (Frank et al., 1985; King and Johnson, 1985), and *in vivo* static measurements of transport (Meijer et al., 2016) suggest the insulin receptor is responsible for physically transporting insulin across the brain endothelial cell and into the brain. However, we have recently shown using dynamic, pharmacokinetic *in vivo* techniques in a mouse model lacking the insulin receptor in brain endothelial cells and use of pharmacological inhibition of the insulin receptor, insulin transport across the BBB is unchanged (Rhea et al., 2018). It was also confirmed by a separate group using primary brain endothelial cells and capillaries from bovine, rat, and mouse that inhibition of the insulin receptor did not affect transport (Hersom et al., 2018). Regional expression of the insulin transporter could be responsible for the regional transport differences. The rationale that the insulin transporter is separate from the insulin receptor is not far-fetched. An important signaling peptide such as insulin should have a protein that it can bind to and elicit an internal signaling cascade in addition to having another protein that can transport this signaling peptide to other areas necessary for signaling (Figure 2). In addition, these separate proteins should be able to be regulated differently, depending on the physiological necessity at any given time. For example, insulin receptor signaling in the brain endothelial cell relays a signal to neurons (Konishi et al., 2017) which we know has multiple effects on growth and survival. In addition, insulin signaling in endothelial cells is a vasoregulator (Vicent et al., 2003). Since endothelial cells create a barrier to other cells within organs, it can be speculated these cells would also contain a transporter to independently get insulin to other cell types in order to act physiologically there. Insulin transport is not related to amino acid transport, the *p*-glycoprotein system, a slow calcium channel, alpha-adrenergic action, or growth hormone, somatostatin, glucagon, or leptin transport (Frank et al., 1985; Banks et al., 1997a; Banks, 2001; Yu et al., 2006). Excess IGF-1 can inhibit the transport of insulin across the BBB, suggesting competitive transport (Yu et al., 2006). However, this study used about a 220-fold excess amount of IGF-1 so the physiological relevance on insulin transport is still unclear. Based on the lack of competitive inhibition with most substrates tested and the data showing changes in insulin transport under various physiological conditions, it is likely that the insulin transporter is rather specific for insulin but is regulated in an allosteric manner.

## Conditions With Altered Insulin BBB Transport

**FIGURE 3:**
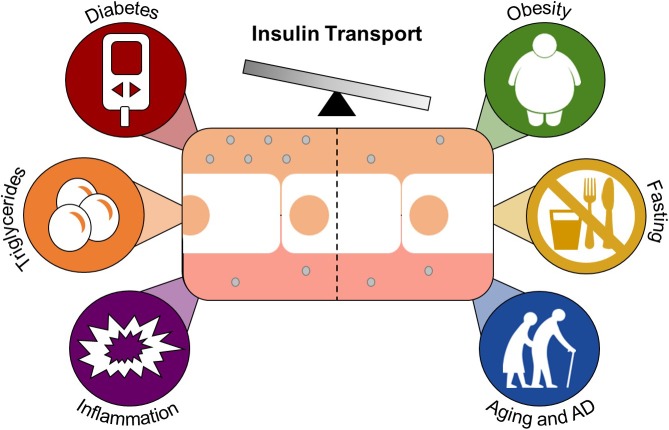
Conditions and factors that regulate insulin transport across the BBB. Brain endothelial cells (center white) separate the blood from the brain and make up the BBB. Insulin (gray circle) is transported across the BBB by a carrier-mediated system. Diabetes, triglycerides, and inflammation are known enhancers of insulin transport. Obesity, fasting, and aging/Alzheimer’s disease attenuate this transport.

While the molecular mediators regulating insulin transport across the BBB are not exactly clear, there are specific conditions and factors that are known to alter the transport rate, total amount, and level of endothelial binding of insulin at the BBB (Banks, 2004). However, since the year of that review (2004), the impact of other factors on BBB insulin transport have been investigated. Estrogen does not impact the transport of insulin into the CSF (May et al., 2016a), while the gastrointestinal hormone cholecystokinin (CCK) increases transport (May et al., 2016b). Other conditions and factors including starvation (Urayama and Banks, 2008), triglycerides (Urayama and Banks, 2008), and nitric oxide (Banks et al., 2008) have also been investigated and will be discussed below with the conditions related to insulin resistance (Figure 3).

### Obesity and Diabetes

Nearly 2 months of high-fat diet feeding in dogs led to an approximate 60% decrease in insulin BBB transport that was inversely proportional to body weight (Kaiyala et al., 2000). Levels of brain insulin were also decreased in obese Zucker rats compared to lean counterparts (Baskin et al., 1985). In addition to overall decreases in brain insulin levels in obese models, it has been shown the transport rate of insulin across the BBB is about half that in obese mice compared to lean mice (Urayama and Banks, 2008). This study also showed starvation (72 h) of obese mice can lead to an increase in transport. However, these changes in transport were eliminated when the contribution of serum factors was removed. This suggests the effect of obesity on insulin BBB transport is mediated by changes in circulating factors, one of which is the triglyceride triolein. Triglycerides increase insulin BBB transport, can cross the BBB, and can induce CNS insulin receptor resistance (Urayama and Banks, 2008; Banks et al., 2018). These data show how a peripheral condition such as obesity can have detrimental effects on CNS insulin signaling.

Contrary to obesity, diabetes mellitus increases BBB insulin transport and endothelial insulin binding, correlating with the onset of diabetes (Banks et al., 1997b). This occurred independent of changes in leptin transport (which share similarities with insulin). Also opposite of obesity, the enhanced transport of insulin across the BBB in the streptozotocin-model of diabetes was not due to changes in serum factors. Instead, there is likely molecular reorganization of the insulin transport system at the BBB due to diabetes. A steady increase in serum glucose following a glucose bolus does not increase the uptake of insulin by the brain but does increase insulin binding to the endothelium (Banks et al., 1997b). These data suggest glucose does not necessarily affect insulin BBB transport but rather can affect interaction with the brain endothelial cell insulin receptor.

Diabetes can also alter the overall structure and function of the BBB. In streptozotocin-treated mice, insulin increases cerebral microvessel expression of tight junction proteins including occludin, claudin-5, and ZO-1 (Sun et al., 2015). Again, this data supports the role of insulin in regulating tight junction protein expression, similar to the data observed in the EndoIRKO mice (Konishi et al., 2017). Expression of the transport protein lipoprotein receptor-related protein 1 (LRP1) is downregulated in mouse brain capillaries (Hong et al., 2009) and CSF LRP1 is increased in type 1 diabetic patients (Ouwens et al., 2014), suggesting insulin may regulate CNS LRP1. Indeed, insulin treatment in an immortalized human brain endothelial cell line increased plasma membrane LRP1 expression but not total cell expression, suggesting a role for insulin in LRP1 translocation (Swaminathan et al., 2018). LRP1 is an important functional regulator within the brain endothelial cell and interacts with multiple substrates, including Aβ, aiding in clearance of this peptide from the CNS. This is one hypothesis as to why people with diabetes are at a higher risk for developing Alzheimer’s disease. LRP1 is also downregulated due to inflammation (Jaeger et al., 2009). Diabetes and obesity can increase inflammation and serum inflammatory mediators, which can have an adverse effect on the BBB.

Inflammation has been shown to have multiple detrimental effects on BBB structure and function. This topic has been extensively reviewed elsewhere (Erickson and Banks, 2018; Van Dyken and Lacoste, 2018). Inflammation due to lipopolysaccharide (LPS) administration enhances insulin BBB transport by up to 2–3 fold (Xaio et al., 2001). Nitric oxide is a signaling molecule in inflammation, released by nitric oxide synthase. Nitric oxide isoenzymes can regulate insulin transport across the BBB under LPS inflammation (Banks et al., 2008). Transcytosis and paracellular transport is increased due to inflammation. Inflammation leads to decreased expression levels of the tight junction proteins claudin-5, ZO-1, and occludin. *P*-glycoprotein, LRP1, and amino acid transporters are also downregulated (Jaeger et al., 2009; Erickson et al., 2012; Ransohoff et al., 2015) while TNF-alpha and Aβ transporters are increased (Jaeger et al., 2009; Varatharaj and Galea, 2017). Proteins responsible for improving transport of immune cells are also increased (Van Dyken and Lacoste, 2018). It is difficult to tease apart the contribution of inflammation, glucose, and changes in hormone levels on BBB permeability in diabetes and obesity. In addition, it is still unclear what the molecular mediators are either at the brain endothelial cell or other cell types of the BBB that alter insulin BBB transport in these various conditions.

### Aging and Alzheimer’s Disease

Due to the data supporting a role for CNS insulin resistance in Alzheimer’s disease, efforts have been made to investigate the transport of insulin in Alzheimer’s disease compared to ‘healthy’ aging. While there was no difference in the rate of insulin BBB transport in an aged non-transgenic Alzheimer’s disease mouse model (SAMP8) compared to young ones, the level of reversible binding at the endothelium was increased regionally (Banks et al., 2000). While this has not been shown molecularly, the level of binding could be considered as a marker for the amount of insulin receptor present. However, in a different mouse model of Alzheimer’s disease (APP/PS1), insulin BBB transport rate is significantly increased in specific brain regions including the hippocampus compared to a wild-type mouse (Poduslo et al., 2001). This could be due to the Aβ interactions with insulin as noted above, as the APP/PS1 mice have higher levels of Aβ that are more fibrillating (and hence more toxic) than the SAMP8. A comparative study utilizing different mouse models of Alzheimer’s disease investigating the transport rate of insulin across the BBB will help us determine what factors might control the rate of transport due to various Alzheimer’s disease pathologies.

There is no disruption of the BBB to serum albumin either in aged SAMP8 mice (Banks et al., 2000) or in APP/PS1 mice that have substantial Aβ levels in the plasma and brain (Poduslo et al., 2001). Instead, there was increased binding of serum albumin, suggestive of a thickened basement membrane. Whether or not BBB breakdown occurs in patients with Alzheimer’s disease is still controversial. A lack of BBB permeability to imaging tracers has been shown in a limited number of subjects (Friedland et al., 1983; Schlageter et al., 1987; Caserta et al., 1998) while others have suggested BBB breakdown precedes pathological hallmarks of Alzheimer’s disease (Nation et al., 2019). The discrepancies reported in the literature could be due to multiple factors including technique used, level of cognitive impairment, or sensitivity of detection.

Patients with Alzheimer’s disease have increased plasma insulin levels, decreased CSF insulin levels, and thus a reduced CSF-to-plasma insulin ratio (Craft et al., 1998). This suggests in Alzheimer’s disease in humans that insulin BBB transport might be impaired. It has also been shown brain insulin levels and insulin receptor levels and signaling are decreased in Alzheimer’s disease (Frolich et al., 1998; Talbot et al., 2012). The insulin immunoreactivity was localized primarily to pyramidal neurons and not glial and endothelial cells. There were also decreases in the insulin receptor expression due to age in the frontal and parietal cortex while expression of the IGF-1 receptor was unchanged suggesting a specific susceptibility.

## Therapies to Increase BBB Insulin Transport and CNS Insulin Levels

In this review, we have summarized the detrimental effects of altered insulin signaling within specific cell types at the BBB. However, many of these detrimental effects are due to decreased exposure to insulin and hence insulin BBB transport. Therefore, if CNS insulin can be increased, some of these detrimental effects could be overcome. Alternative routes to increase CNS insulin has recently been reviewed by our group in detail (Rhea et al., 2019). We have highlighted here a couple of therapies that are the most translational to increase CNS insulin levels.

### Intranasal

Intranasal insulin can improve memory in young, healthy adults (Benedict et al., 2004), people with mild cognitive impairment and Alzheimer’s disease (Reger et al., 2008a,b), and in mouse models of Alzheimer’s disease (Salameh et al., 2015; Mao et al., 2016). When insulin is delivered intranasally, it reaches most brain regions in both young and aged wild-type and SAMP8 mice (Salameh et al., 2015; Rhea et al., 2017). This therapy has been shown to improve parts of insulin receptor signaling in the forebrain (Chen et al., 2014) and hippocampus (Mao et al., 2016). It is possible insulin acts independent of the insulin receptor to improve memory. One potential mechanism could be via the interaction of insulin and Aβ. Intranasal insulin is able to reduce Aβ plaque deposits by altering the processing of the APP peptide (Mao et al., 2016). Even though insulin signaling in the CNS cannot be studied in living humans, brain slices taken post-mortem from patients with Alzheimer’s disease respond to *ex vivo* insulin stimulation (in terms of insulin receptor signaling phosphorylation), but not at the level of age-matched controls (Talbot et al., 2012). This suggests that while insulin signaling is impaired in Alzheimer’s disease, the brain still has the ability to respond to insulin. Therefore, delivering insulin to the site of action as occurs with intranasal delivery will help to overcome CNS insulin resistance.

### Weight Loss

Since obesity is commonly linked with insulin resistance, weight loss should restore insulin sensitivity. As mentioned earlier, in animal models of obesity, insulin transport across the BBB is impaired (Schwartz et al., 1991; Urayama and Banks, 2008). In an animal study investigating the response of BBB insulin transport to weight loss, CSF insulin levels increase following diet reversal and weight loss in male Long-Evans rats (Begg et al., 2013). The rats were on a high-fat diet for 22 weeks before a group of them were switched to a low-fat diet for 8 weeks. Therefore, diet-induced CNS insulin resistance can be reversed if switched to a low-fat diet. Weight loss is associated with a decrease in triglyceride levels. Triglycerides increase insulin BBB transport (Urayama and Banks, 2008) but can cross the BBB and impair CNS insulin signaling (Banks et al., 2018). It is unclear whether diet reversal can improve CNS insulin resistance in humans. One could measure CSF and serum insulin levels following weight loss to assess potential changes in transport. Indeed, weight loss is associated with improved cognition (Veronese et al., 2017). Triglycerides and other lipids including cholesterol and omega-3 fatty acids have an impact on memory and cognition (Morley and Banks, 2010). Studies investigating changes in CNS insulin sensitivity following dietary, behavior changes or surgery, such as bariatric surgery or liposuction, would help elucidate the true impact of weight loss on insulin BBB transport.

## Conclusion

Here, we have summarized for the condition of insulin resistance the interplay of the BBB and various types of other brain cells that form the NVU. This interplay has emerged to be particularly important in Alzheimer’s disease and provides mechanisms for specific interactions with Aβ and tau and links with metabolic disease. The BBB with its transport system for insulin is critical for defining the interactions between peripherally secreted insulin with its receptors located within the CNS. Thus, the BBB is also critical in considering therapeutic options. Overall, the barrier functions in combination with the communication functions of the BBB result in an operational blood-brain interface vital to understanding insulin resistance.

## Author Contributions

ER established the review topic. ER and WB wrote and edited the manuscript.

## Conflict of Interest Statement

The authors declare that the research was conducted in the absence of any commercial or financial relationships that could be construed as a potential conflict of interest.

## Footnotes

1 http://www.brainrnaseq.org/
